# Xylem growth cessation in stems and branches of European beech and silver birch: influences of temperature and drought

**DOI:** 10.3389/fpls.2025.1648689

**Published:** 2025-08-26

**Authors:** Lorène J. Marchand, Jožica Gričar, Peter Prislan, Inge Dox, Melanie Verlinden, Omar Flores, Matteo Campioli

**Affiliations:** ^1^ Plants and Ecosystems (PLECO) Research Group, University of Antwerp, Antwerp, Belgium; ^2^ Department of Forest Physiology and Genetics, Slovenian Forestry Institute, Ljubljana, Slovenia; ^3^ Department for Forest Technique and Economics, Slovenian Forestry Institute, Ljubljana, Slovenia; ^4^ Earth Sciences, Vrije Universiteit Amsterdam, Amsterdam, Netherlands

**Keywords:** phenology, cambium, cell wall thickening, hardwood species, atmospheric drought

## Abstract

**Introduction:**

Assessing wood growth phenology over multiple years is essential for understanding the environmental drivers of forest growth and improving large-scale predictions of the carbon cycle. Xylogenesis methods facilitate the assessment of the timing and rate of xylem cell wall thickening, the primary sink of carbon in wood. In angiosperm trees, where wood anatomy is complex, significantly less is known about the factors controlling growth cessation in autumn due to indirect, sinteracting, and lag effects, in contrast to growth resumption in spring. Furthermore, both branch and stem growth must be considered to account for the total aboveground phenology.

**Methods:**

In this study, we focused on European beech (*Fagus sylvatica*) and silver birch (*Betula pendula*) in a mild temperate region (Northern Belgium). We examined the progress of cell wall thickening in autumn and the seasonal timing of xylem growth cessation for these species’ stems over five years and for their branches over one year in mature trees. In addition, we investigated the same variables in the stems and branches of potted saplings for two years and for oak (*Quercus robur*) and aspen (*Populus tremula*) saplings over one year.

**Results:**

Our results demonstrate a considerable variation in the progression and cessation of wood growth, with differences of up to a month and a half in growth cessation (early September to late October), predominantly driven by climatic variables. Early cessation of xylem growth in stems was strongly associated with high temperatures in April and August, elevated vapour pressure deficit, and severe soil drought in August. The progression of cell wall thickening in late summer was generally synchronized between branches and stems for every species. However, branches sustained a higher percentage of growth (approximately 2 weeks) in early autumn during non-drought years.

**Discussion:**

These findings provide valuable insights for refining models of forest growth and carbon storage, enabling a more comprehensive representation that encompasses the entire tree under different climatic scenarios.

## 1 Introduction

For trees, the most noticeable phenological events are related to leaves (e.g. bud-burst), flowers (e.g. blooming) or fruit (e.g. fruit maturation) (Campioli et al., 2025). Phenological events related to wood growth, involving the production of xylem (xylogenesis) and phloem cells, are less visible but equally important. Xylogenesis involves the production, enlargement, and secondary cell wall thickening of new xylem cells, which provide mechanical support or conduct water after maturation (cell death) (Rathgeber et al., 2016). Xylogenesis starts concurrently with or before leaf phenology (Marchand et al., 2021). The first mature xylem cells are usually observed in June in temperate forests (Prislan et al., 2019; Dox et al., 2020). As autumn approaches, cell production ceases in late summer and cell enlargement ceases quickly afterwards, while cell wall thickening typically continues for 4 to 10 weeks in temperate forests (Prislan et al., 2019; Dox et al., 2020; Gričar et al., 2022). Cell wall thickening contributes to 90% of the biomass and carbon accumulation in wood (Cuny et al., 2015). However, cell wall thickening dynamics cannot be accurately observed with the commonly used dendrometer methods, which only record the enlargement process (Cuny et al., 2015). Therefore, to model carbon sequestration in wood and the carbon cycle more broadly, deeper knowledge of a key parameter, such as cell wall thickening dynamics, is needed (Eckes-Shephard et al., 2022). While efforts have been made to investigate the onset of cell wall thickening, its cessation remains elusive (Prislan et al., 2019; Dox et al., 2022a).

The timing of the cessation of cell wall thickening is challenging to elucidate due to the potential interplay of current conditions (late summer–early autumn) and intra-seasonal legacy effects, which may also be influenced by extreme climatic events during summer (Dox et al., 2021, 2022a; Gričar et al., 2022). The scarcity of long-term time series further complicates this analysis, largely because such data require intensive and sustained sampling efforts (Silvestro et al., 2024). For angiosperms, only a few time series spanning only three years have been documented (Martinez del Castillo et al., 2016, 2018; Chen et al., 2022; Dox et al., 2022b). Temperature is evidenced as a key driver of autumn leaf- and xylem phenology, not only summer temperature but also spring temperature acting as a legacy effect (Prislan et al., 2019; Zohner et al., 2023). Moreover, summer drought can advance the cessation of xylem growth by several weeks in deciduous trees, especially for late-successional species (Dox et al., 2021, 2022a; Gričar et al., 2022). With climate change expected to increase the frequency and severity of summer droughts in temperate regions, it becomes important to investigate how both spring and summer meteorological conditions influence xylem growth cessation (Mariën et al., 2021a; Martinez del Castillo et al., 2022).

This research should extend beyond the stem to include other woody organs, such as branches, which serve for the transport and temporary storage of photoassimilates between the canopy and the trunk (Zahnd et al., 2023, 2024). Depending on tree size and age, branches can constitute up to 30% of the total aboveground woody biomass because of their high wood density (André et al., 2010; MacFarlane, 2020). Very few studies compared the xylogenesis of stem and branches, especially in the second half of the growing season. Gričar et al. (2017) found that cell wall thickening in branches of mature *Quercus pubescens* Willd. in central Europe ceased 2–3 weeks earlier than in the stem. This basipetal xylem growth cessation goes in the same direction as reserve content flow in autumn (Zahnd et al., 2023). However, it remains uncertain whether this pattern is valid across tree species or how it varies. Elucidating these aspects will greatly help to understand the aboveground growth dynamics at the end of the growing season in temperate species.

In this study, we tested two hypotheses: (i) warm spring and summer drought advance the timing of the cell wall thickening cessation in deciduous angiosperm species and (ii) in autumn, branches exhibit reduced activity of xylem cell wall thickening compared to the stem. Our study focused on two of the most common species of angiosperm deciduous forest trees of the temperate European zone: European beech (*Fagus sylvatica* L.) and silver birch (*Betula pendula* Roth.). These species differ in successional stage (with birch being a pioneer while beech late-successional species). Our study relies on data of stem xylogenesis in the second half of the growing season over five years for mature birch and beech in semi-natural stands, as well as parallel measurements of branch xylogenesis on mature trees (one year) and potted saplings (two years) of the two species. To further enhance our understanding of the coupling between branches and stems, we also present cell wall thickening data on both organs for potted saplings of pedunculate oak (*Quercus robur* L.) and common aspen (P*opulus tremula* L.), with oak being a late-successional and ring-porous species, while aspen is a pioneer and diffuse-porous species.

## 2 Materials and methods

### 2.1 Sampling for xylogenesis - mature trees

The forest stands were located near Antwerp (Belgium) within Klein Schietveld in Kapellen (51°21′ N, 4°37′ E) for birch and in the Park of Brasschaat in Brasschaat (51°12′ N, 4°26′ E) for beech. The distance between the stands is 7 km, with flat topography. The area is characterized by a temperate climate, with a mean annual temperature of 11°C and a mean annual precipitation of 808 mm over the past 30 years (1992–2021). The average temperature and accumulated precipitation in mid-summer (July–August) are 17.8°C and 227 mm, while the same variables in mid-spring (April and May) are 11.9°C and 101 mm, respectively. The soil in the region is generally sandy and nutrient-poor. However, the beech site has a deep anthropogenic organic layer typical of this region with a long history of cultivation, where substantial amounts of mineral and organic fertilizers were applied, or where materials such as sods or shells were regularly added to the land (Dondeyne et al., 2014; Mariën et al., 2021b).

Each year between 2017 and 2021, we selected (co)dominant mature birch and beech trees (n = 4–8 per species, depending on the year) to study xylogenesis in the late season. The mean diameter and height of the studied beech trees were 51 ± 4 cm and 24 ± 1 m, respectively. For birch, these values were 24 ± 1 cm and 20 ± 0.4 m, respectively. The age of the trees was approximately 70 years for beech and 45 years for birch, based on coring performed for a previous study (Marchand et al., 2020). The stands have been extensively monitored for leaf, stem, and root autumn phenology since 2017 (Mariën et al., 2019, 2022; Dox et al., 2020, 2022b; Marchand et al., 2025).

Each year, between early August and mid-November, stem micro-cores were collected weekly from each tree following a helicoidal pattern starting at breast height, using a Trephor tool (Rossi et al., 2006; Dox et al., 2020). Branch sampling was exploratory and time- and resource-consuming; it was only successfully conducted in 2020. In 2020, between early August and mid-November, micro-cores of the three main branches per tree (on the four replicate trees per species) were collected twice a month by tree climbers using a Trephor tool, following a helicoidal pattern. The branches were located in the central part of the crown and were sampled at a distance of 1.5–2 m from the branch apex, where the branch diameter was approximately 5–10 cm.

### 2.2 Sampling for xylogenesis – saplings

The sapling pot experiment took place in a common garden at the Drie Eiken Campus in Wilrijk, Belgium (51°09′ N, 4°24′ E), located approximately 20 km from the forest stands. In 2017 and 2018, we purchased 2–3-year-old beech and birch saplings of local provenance from a local nursery (n = 48 per species). In addition, in 2017, 3-year-old oak and 1-year-old aspen sampling were purchased too from the local nursery (n=48 per species). Saplings were planted in February of each year into 35 L pots filled with a nutrient-poor substrate (90% of fine sand and 10% peat by soil volume). An optimal amount of slow-release fertilizer (70 g NPK, plus micro-elements) and chalk (30 g) was added. The pots were displayed in square plots, each plot containing 16 pots (4 x 4). The spaces between and around the pots were filled with soil to provide thermal insulation. During the growing season, the trees were generally irrigated every 2–3 days, but more frequently during warm and dry periods. The experimental site was kept for only 2 years due to the destructive nature of the sampling.

From late August to the end of November in 2017 and 2018, 3–5 saplings per species were harvested every 1–2 weeks (n = ~160 saplings). On each harvested sapling, a stem micro-core was sampled, and one (2017) or three (2018) non-apical branches were collected, from which a section (approximately 5–10 mm in diameter and 2–3 cm in length) was taken (one section per branch). We increased sampling to three branches in 2018 to account for potential branch variability in xylogenesis. In 2017, stem sampling started only from mid-September to limit the destruction of saplings at the start of the experiment. Furthermore, in 2017, the same sampling was also performed on saplings of oak and aspen in the same way as beech and birch.

### 2.3 Meteorological data

For the years 2017 to 2021, air temperature and vapor pressure deficit (VPD) above the canopy (at 32 m) were available from a meteorological station (ICOS and ICP Forest) at hourly resolution (Gielen et al., 2017). Soil water content (SWC) at 0–10 cm, 10–20 cm, 20–40 cm and 40–80 cm depth (average of the four depths was calculated, measured 4 times a day) was also available from the same meteorological station (De Vos et al., 2021). The weather station was approximately 6.5 km from the mature forest stands, on very similar soil but in a mixed stand (Janssens et al., 1999). We used the Standardized Precipitation Evapotranspiration Index on a 1-month average (SPEI) as a multiscalar drought index for the area (Vicente-Serrano et al., 2010). SPEI computation comes from the SPEI data repository https://spei.csic.es/using where precipitation and evaporation differences are used to estimate the water balance. We tested monthly meteorological conditions in spring (April and May) and summer (June, July and August) as potential drivers of xylem cessation timing across years based on findings of previous xylogenesis and senescence studies (Prislan et al., 2019; Zohner et al., 2023). Average maximal temperature, VPD, SPEI, and median SWC were studied.

### 2.4 Leaf phenology

Leaf phenology was assessed weekly, from late August to mid-November, at the individual tree level for mature trees over 2017–2021 and on a subset of the saplings (n=12) for both species and years (2017 and 2018). We visually estimated the percentages of leaves that changed color and the percentage of leaves that had fallen (Mariën et al., 2019). These data were combined following Vitasse et al. (2009).

### 2.5 Wood lab analyses

**Figure 1:**
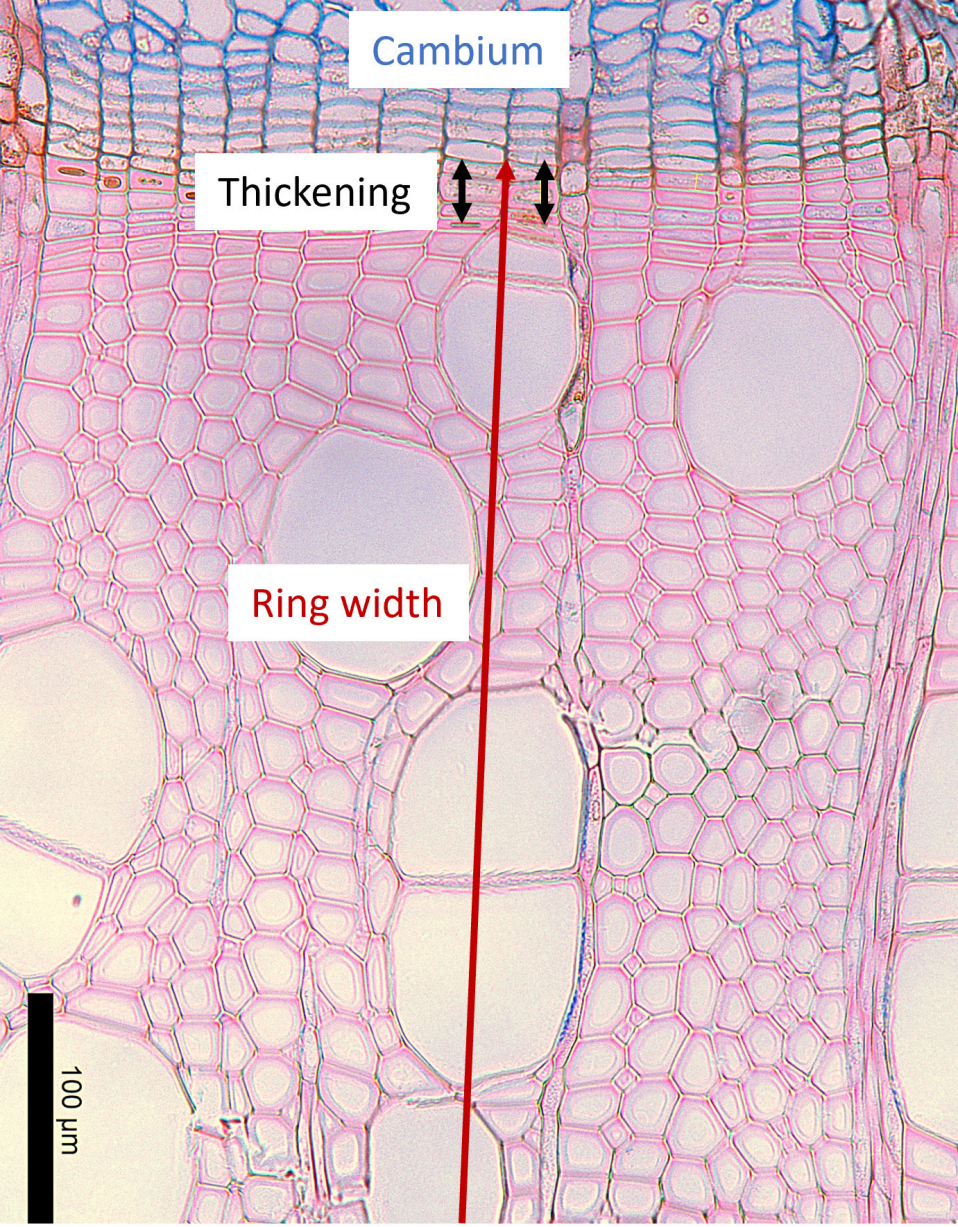
Microscopic section of silver birch (*Betula pendula*) prepared near the end of the growing season, on 29 September 2019, showing a few xylem cells still undergoing secondary wall thickening. The section was stained with Astra Blue and Safranin. The cambial zone, stained blue, is visible at the top of the image; the last differentiating cells in the wall thickening phase are marked in black; and the developing annual ring (partially visible) is outlined in red.

All micro-cores and cross-sections were stored in an ethanol (70%) solution. Samples were prepared in slides following the protocol of Prislan et al. (2022) and observed under a Leica DM 400 B/M light microscope with Leica LAS image analysis system (Leica Microsystems, Wetzlar, Germany). In angiosperms, cell counting is not feasible in the same way as in gymnosperms due to the irregular arrangement and lack of alignment of xylem cells. Therefore, we used the protocol developed by Dox et al. (2020) for angiosperm woody tissue, to define for each sampling date the percentage of the width of xylem cells undergoing cell wall thickening on the width of xylem cells produced in the current year (**Figure 1**). This was derived by measuring the current ring width and the width of the layer of cells undergoing wall thickening. For each tree or sapling, the data were the mean of three replicate measurements on the sample (**Figure 1**) (Gričar et al., 2017; Dox et al., 2020). The timing of xylem growth cessation (in DOY) (of relevance here only for mature trees) was defined at the tree level as the date when the proportion of the xylem cell wall thickening in the current ring was lower than 1% for at least 3 consecutive sampling dates. The average wood growth cessation per year was calculated. Due to broken samples, some measurements were not done, which concerned only 4 stem samples (1 betula and 1 fagus in 2018 and 1 fagus in 2020) and 7 branches ( 3 betula and 1 fagus in 2018 and 3 fagus in 2020).

### 2.6 Statistics

All statistics were performed using R v. 4.3.1 (R Core Team, 2024) and graphics were built with packages ggplot2 (Wickham, 2016), ggrepel (Slowikowski et al., 2024) and ggpubr (Kassambara, 2023). Comparison of the climatic variables (e.g. April-June T-max, June-August SWC) across the five studied years was made with a Dunn test, two-sided with Benjamini-Hochberg correction to control for the false discovery rate, except for SPEI due to the smaller dataset (functions used: dunnTest()from package ‘FSA’ (Ogle et al., 2025) and cldList() from ‘rcompanion’ (Mangiafico, 2024)). This test was done on the mean for SPEI, Tmax and VPD, but on the median for SWC as the SWC mean did not meet the required assumption of symmetric data distribution. Dunn test, but without correction (due to the smaller dataset), was used to compare the timing of stem xylem cessation across years. To study the relation between stem xylem cessation and climatic variables, linear models were performed using lm() function in the ‘stats’ package (R Core Team, 2024). The prerequisites were checked and met. The comparison of the percentage cell wall thickening between stem and branches per date was done by applying a Fligner-Policello test (fp.test() function in the RVaidememoire package; Hervé, 2016).

## 3 Results

### 3.1 Spring and summer climatic conditions

**Figure 2:**
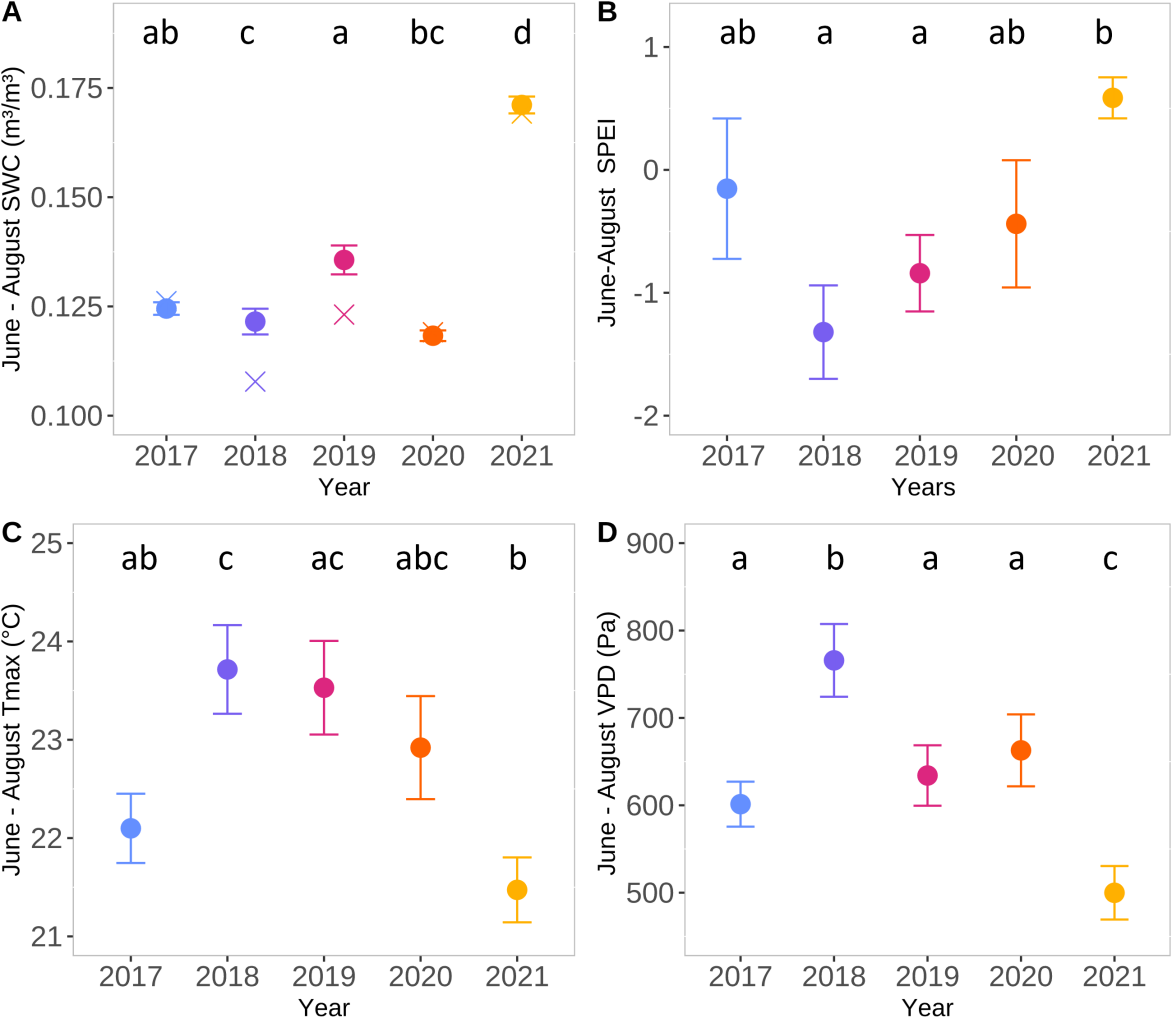
Meteorological conditions from June to August for the period 2017–2021 at Brasschaat. Average of soil water content (SWC) (m²/m³) at 0-80cm depth **(A)**, Standardised Precipitation Evapotranspiration Index (SPEI) **(B)**, daily maximal air temperature (Tmax; °C) **(C)** and vapour pressure deficit (VPD; Pa) **(D)** during June, July and August at Brasschaat over the five years studied. For SWC also the median is shown. Different letters indicate significant differences between years based on the Dunn test. The points indicate the mean and the crosses the median. Color code: 2017 in blue, 2018 in purple, 2019 in pink, 2020 in orange and 2021 in yellow. Daily values of SWC, Tmax and VPD are presented in **Supplementary Figure 1**.

The weather during the peak growing season (June–August) of 2018 was exceptionally dry and warm, making it one of the warmest and driest summers in the past 30 years (1992–2021; Mariën et al., 2021a). It featured, in particular, significantly higher VPD and lower SWC in summer than the other years analyzed (**Figure 2**; **Supplementary Figure 1**). However, the spring (April–May) maximum temperature in 2018 was not significantly different from that in 2019 or 2020 (**Supplementary Figure 2**). In contrast, the summer of 2021 was wetter and cooler than the other four summers (**Figure 2**; **Supplementary Figure 1**). Additionally, the spring of 2021 was the coolest (**Supplementary Figure 2**). As in 2018, the years 2017, 2019, and 2020 were also drier and warmer than the long-term average (especially due to various summer heatwaves; described in Mariën et al., 2021a, 2022). However, they were less extreme than 2018, with values of Tmax, VPD, SWC, and SPEI falling between those of 2018 and 2021 (**Figure 2**; **Supplementary Figure 1**).

### 3.2 Cell wall thickening and xylem growth cessation in stem for mature trees in 2017-2021

**Figure 3:**
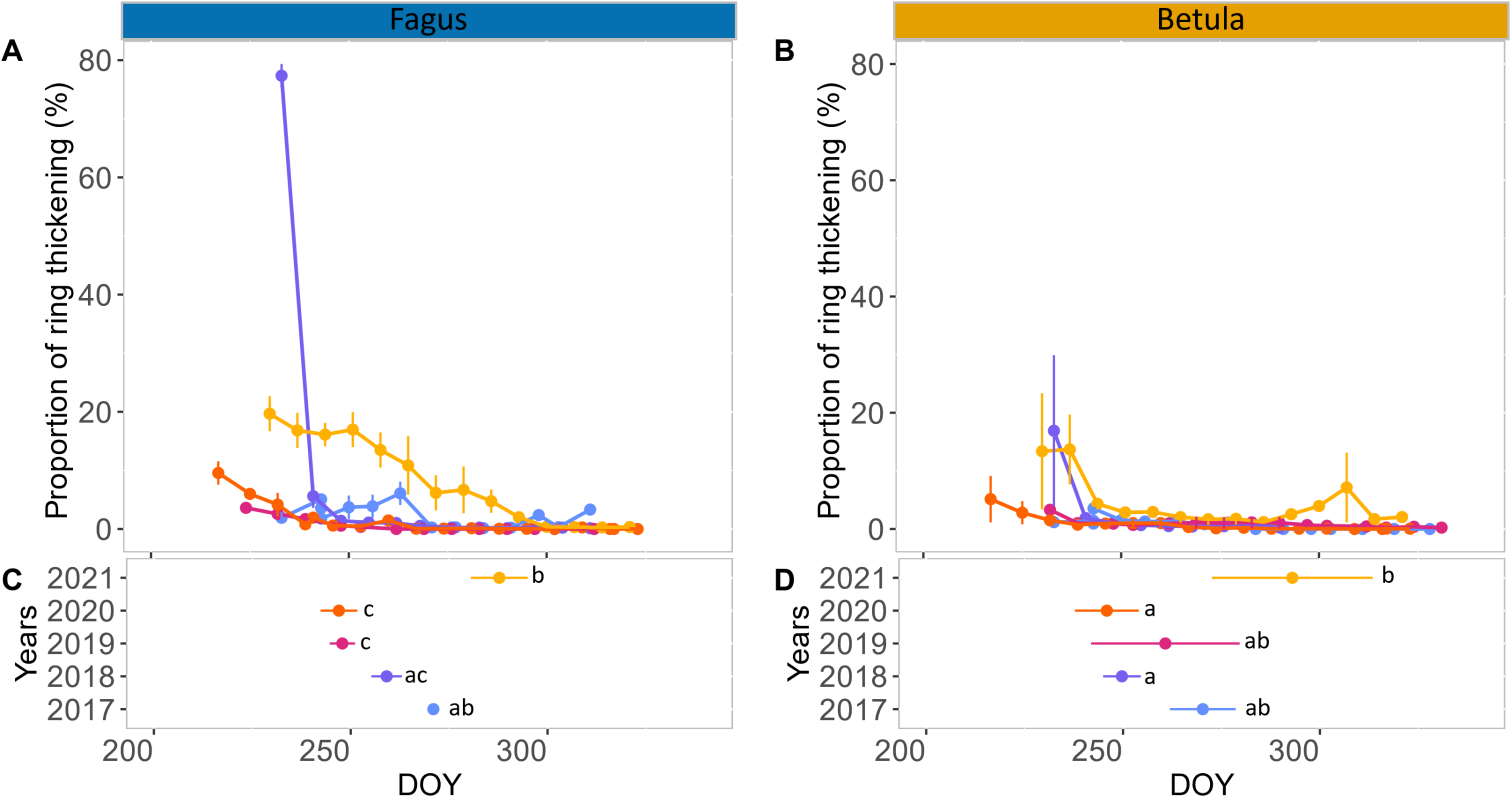
Xylem stem ring wall thickening in autumn. Proportion of stem xylem ring wall thickening (mean across trees and standard error per date) in the late summer and autumn of 2017–2021 for mature trees of European beech (*Fagus sylvatica* L.) **(A)** and silver birch (*Betula pendula* Roth.) **(B)**. Mean and standard error of the timing of xylem stem cessation for mature trees of beech and birch are shown in **(C, D)**, respectively. Different letters indicate significant differences in the timing of xylem stem cessation between years based on the Dunn test. Color code: 2017 in blue, 2018 in purple, 2019 in pink, 2020 in orange and 2021 in yellow.

**Figure 4:**
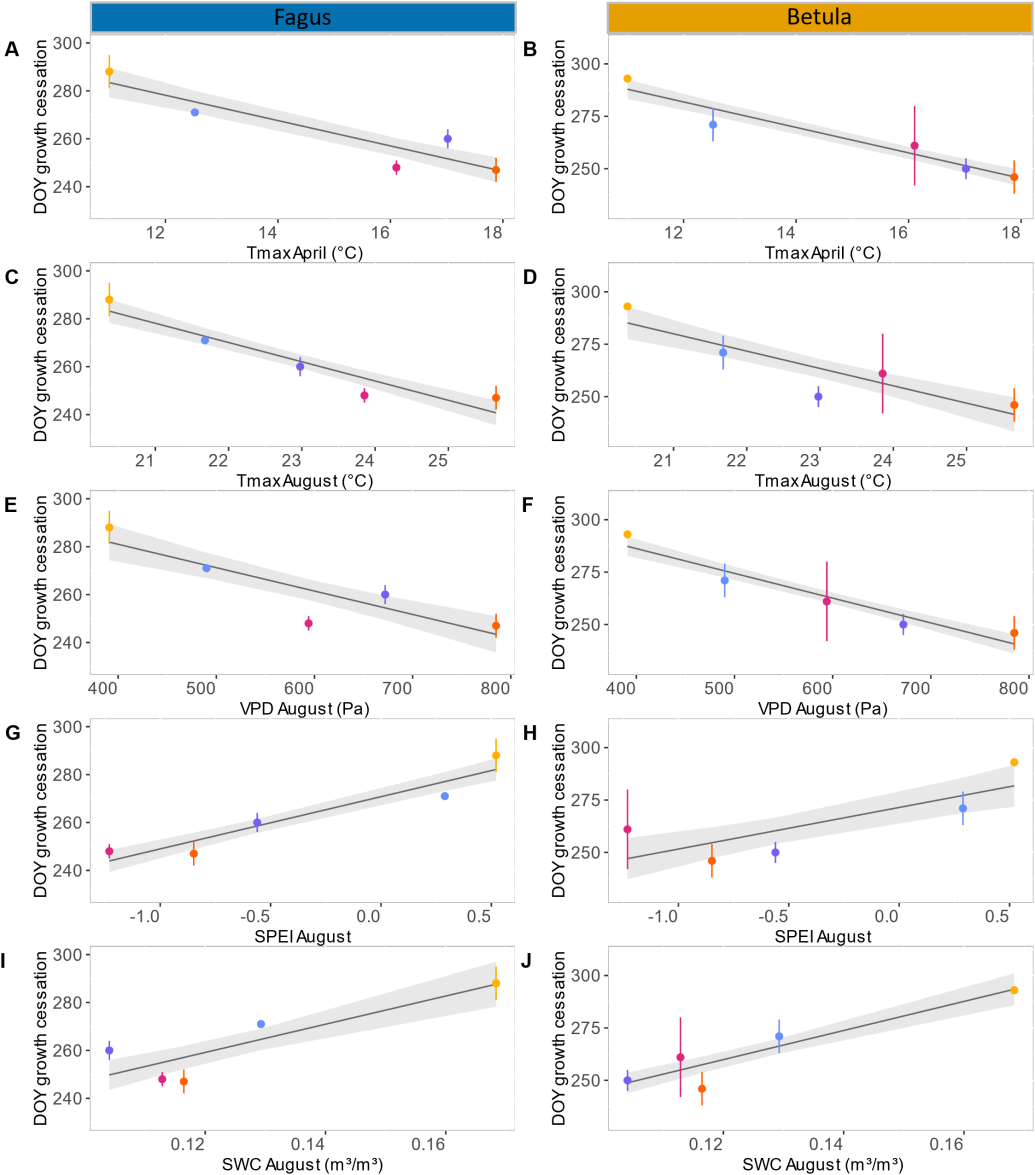
Relatiosnhip between stem xylem growth cessation timing and climatic variables: linear models of the timing of cessation of stem xylem growth in relation to the mean of daily maximal temperatures (Tmax) in April for beech **(A)** and birch **(B)**, the mean of daily maximal temperatures (Tmax) in August for beech **(C)** and birch **(D)**, the mean of daily vapor pressure deficit (VPD) in August for beech **(E)** and birch **(F)**, Standardized precipitation evapotranspiration index (SPEI) in August for beech **(G)** and birch **(H)**, and the median soil water content at 0-80cm depth (SWC) in August for beech **(I)** and birch **(J)**. Color code: 2017 in blue, 2018 in purple, 2019 in pink, 2020 in orange and 2021 in yellow. The black line represent the linear model and the grey bands represent the confidence interval of 95%.

In both 2019 and 2020, seasonal xylem cell wall thickening in the stems of birch and beech followed a similar pattern. At the start of monitoring in late August, only a small proportion (maximum 10%) of the xylem ring exhibited cell wall thickening, which rapidly declined to zero after a few weeks (**Figures 3A, B**; **Supplementary Table 1**). This resulted in xylem growth cessation between DOY 246 and 261. In 2017, we observed a small (<10%) and relatively constant proportion of xylem ring cell wall thickening until late September, which resulted in xylem growth cessation around DOY 270. This pattern was particularly clear for beech but also occurred (with lower values) for birch (**Figures 3C, D**; **Supplementary Table 1**). In 2018, for both species, the proportion of xylem ring cell wall thickening was high at the first sampling in late August but abruptly declined afterwards, reaching growth cessation at DOY 250–260. In 2021, xylem cell wall thickening continued for a long time, with growth cessation around DOY 290 (**Figures 3C, D**; **Supplementary Table 1**). During this year, the decline in xylem cell wall thickening was very gradual for beech (from approximately 20% to 0% in two months), while for birch the proportion of xylem ring cell wall thickening remained small (approximately 2%) for most of September and October (**Figures 3A, B**; **Supplementary Table 1**). Overall, individual variability of xylem growth cessation was greater for birch than for beech (**Figures 3C, D**; **Supplementary Table 1**). Among the summer climatic variables characterizing June, July, and August, only the conditions in August showed a significant relation to the timing of xylem growth cessation (though, for birch, also July SWC was significant; **Table 1**). Similarly, among the spring climatic variables of April and May, only April exhibited a significant correlation with xylem growth cessation timing (**Table 1**). More precisely, for birch, the mean VPD in August and mean Tmax in April could explain 90% of the variability of the xylem growth cessation timing across years. Median SWC in August and mean Tmax in August could also explain a large portion of this variability (70 – 80%) (**Table 1**; **Figure 4**). For beech, SPEI in August, Tmax in August and Tmax in April could explain 80-85% of the xylem growth cessation timing differences across years. Median SWC in August was almost significant (p-value= 0.06) and could explain 67% of the variability (**Table 1**; **Figure 4**).

**Table 1:** Relationship between stem xylem growth cessation and climatic variables.

climate variables	Fagus			Betula		
	T- value	P-value	R² adj.	T- value	P-value	R² adj.
Mean April Tmax	-4.056	**0.027**	79	-6.177	**0.008**	90
Mean May Tmax	-0.401	0.715	0	-1.294	0.286	14
Mean June Tmax	0.496	0.654	0	1.425	0.249	20
Mean July Tmax	-0.500	0.651	0	-0.853	0.456	0
Mean August Tmax	-5.102	**0.015**	86	-3.346	**0.044**	72
Mean June VPD	0.762	0.501	0	0.800	0.482	0
Mean July VPD	-0.715	0.526	0	-1.257	0.298	12
Mean August VPD	-3.101	**0.053**	68	-6.177	**0.009**	90
Mean June SPEI	0.048	0.964	0	0.427	0.698	0
Mean July SPEI	2.076	0.13	45	0.056	0.056	67
Mean August SPEI	5.20	**0.013**	87	2.21	0.114	50
Mean June SWC	0.028	0.978	0	0.402	0.714	0
Mean July SWC	2.517	0.086	57	3.632	**0.036**	75
Median August SWC	2.996	0.06	67	4.394	**0.021**	82

Output of the univariate linear models of the timing of stem xylem growth cessation of Betula (*Betula pendula*) and Fagus (*Fagus sylvatica*) in relation to current year meteorological variables. Tmax, maximum temperature; VPD, vapor pressure deficit; SPEI, Standardised Precipitation Evapotranspiration Index; SWC, soil water content at 0–80 cm depth. N=5 for both Betula and Fagus; bold indicates p-value <0.05. R² adj: adjusted R² (in percentage) for the linear models.

### 3.3 Branch- and stem cell wall thickening in mature trees in 2020 and saplings in 2017

**Figure 5:**
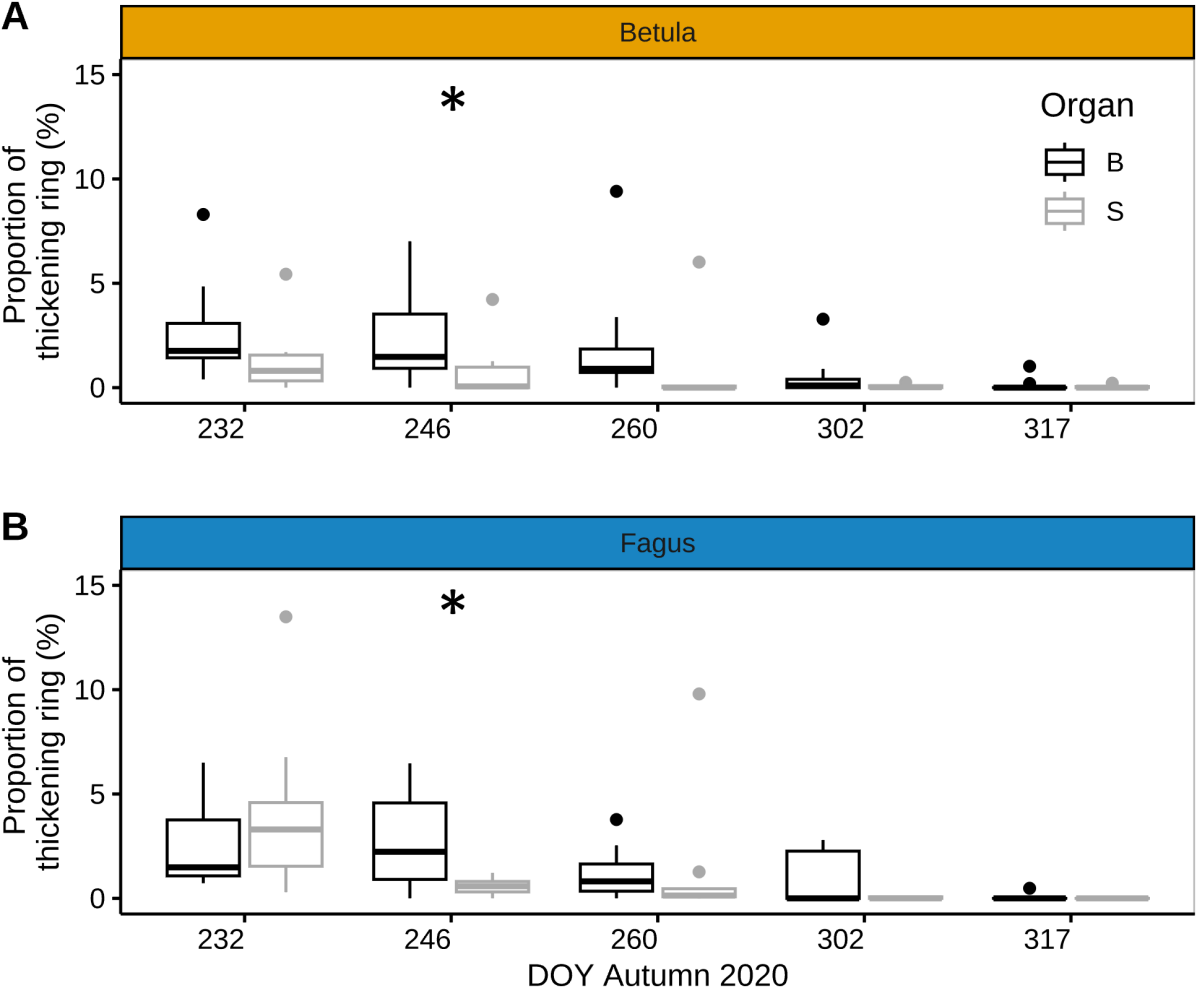
Xylem ring wall thickening in stem and branches of mature trees in late summer - autumn. Proportion of xylem ring wall thickening in late summer - autumn 2020 for branches (black) and stem (grey) for mature individuals of European beech (*Fagus sylvatica* L.) **(A)** and silver birch (B*etula pendula* Roth.) **(B)** (n=8 trees per species for stem and n=4 trees per species for branches). Symbols: bold line: median; box: interquartile range; vertical lines: minimum and maximum values, and dots: outliers. Significant difference between branch- and stem ring thickening (based on Fligner Policello test) is indicated with ‘*’ (p-value < 0.05).

In late summer and autumn of 2020, the difference in the proportion of xylem ring cell wall thickening between the stem and branches of mature trees was non-significant, except for one sampling date in early September (DOY 246), when branches displayed significantly higher xylem ring cell wall thickening than the stem for both species (**Figure 5**). This was because, between late August and early September, xylem ring cell wall thickening in branches did not decrease (beech) or decreased less (birch) than in the stem during the same period (**Figure 5**).

**Figure 6:**
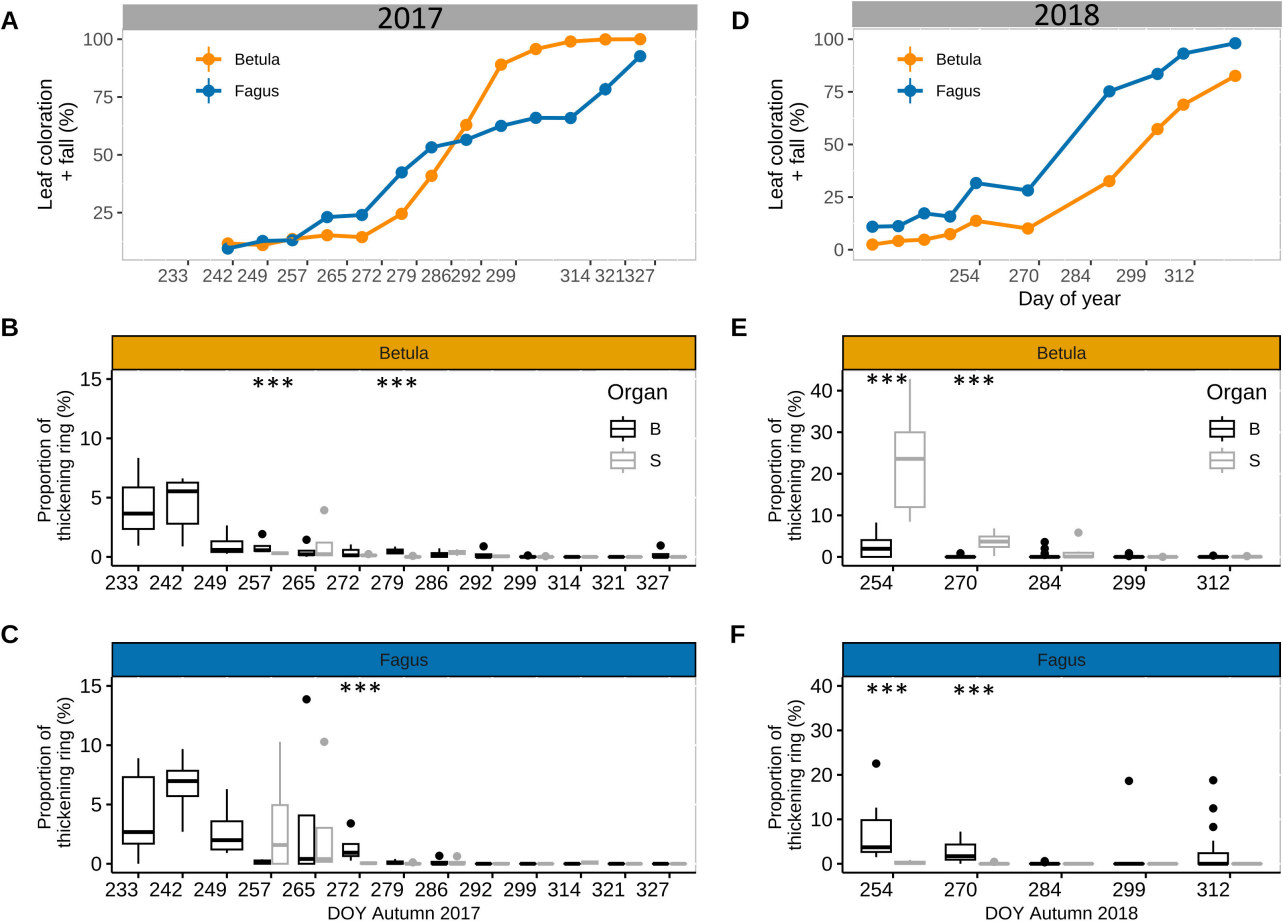
Stem and branch xylem ring wall thickening in late summer-autumn for saplings. Leaf senescence (proportion of yellow and fallen leaves) in autumn 2017 **(A)** and autumn 2018 **(D)** for saplings of silver birch (*Betula pendula* Roth.) (orange) and European beech (*Fagus sylvatica* L.) (blue) (n = 12 sapling per species). Proportion of xylem ring wall thickening of branches (B, black) and stem (S, grey) in later summer - autumn for birch [2017 **(B)** and 2018 **(E)**] and beech [2017 **(C)** and 2018 **(F)**] (n=5 trees per species). Symbols: bold line: median; box: interquartile range; vertical lines: minimum and maximum values, and dots: outliers. Significant differences between branch- and stem ring thickening (based on Fligner Policello test) is indicated with ‘***’ (p-value < 0.001).

For saplings, we observed a consistent seasonal trend in cell wall thickening of the stem and branches, as measured for mature trees. In late summer and autumn of 2017, cell wall thickening decreased over time for both branches and the stem in both beech and birch (**Figures 6B–F**), parallel to the progression of the senescence process (**Figures 6A, D**). For branches, cell wall thickening was still moderate (3–7%) in late August–early September but then decreased rapidly afterwards, especially for birch. In fact, for the branches of the latter species, values of cell wall thickening <1% were already observed in mid-September, while for beech these values were observed in early October. For the stem, in mid-September, cell wall thickening still occurred for beech (3.4 ± 2.4%) but was practically zero for birch (0.3 ± 0.06%). Overall, across the 10 sampling events in 2017, differences in cell wall thickening proportions between the stem and branches were non-significant, except in 3 cases with larger values in branches than in the stem for birch (in mid-September and mid-October) and beech (in mid-October) (**Figures 6B–E**). These significant differences were small (with a maximum difference of 1.4% vs 0.06% in cell wall thickening). Data for aspen and pedunculate oak confirmed the pattern observed for beech and birch, but without showing any significant differences in cell wall thickening proportions between the stem and branches in late summer and autumn (**Supplementary Figure 3**).

### 3.4 Comparison branch- and stem cell wall thickening of saplings in 2017 and 2018

For birch in 2018, the proportion of cell wall thickening in the stem in mid- and late September was 23.1 ± 4.9% and 3.6 ± 1.2%, respectively, which was significantly greater than in 2017 (t-value = -3.3; p-value = 0.009; **Figures 6B, E**). Conversely, the proportion of cell wall thickening in the branches during the same period in 2018 was 2.4 ± 0.6% and 0.07 ± 0.07%, respectively. These values were comparable, to some extent, to the corresponding values in 2017 (t-value = -2.0; p-value = 0.06; **Figures 6B, E**). As a result of these patterns, the proportion of cell wall thickening in September 2018 was significantly greater in the stem than in the branches (**Figure 6D**). Overall, we observed a longer cell wall thickening activity in the stem in 2018 than in 2017, as it stayed above the 1% threshold until DOY 284, while in 2017 this threshold was already reached by DOY 265 (**Figures 6B, E**). The progression of leaf senescence was also slower in 2018 than in 2017 (**Figures 6A, D**).

For beech in 2018, the proportion of cell wall thickening in the stem in mid- and late September was 0.3 ± 0.2% and 0.09 ± 0.08%, respectively, which was comparable to the values in 2017 (t-value = 1.66; p-value = 0.12; **Figures 6C, F**). In the same period, the proportion of cell wall thickening in the branches was 6.7 ± 1.7% and 2.6 ± 0.5%, respectively, also comparable to the values in 2017 (t-value = -0.05; p-value = 0.09; **Figures 6C, F**). Thus, the proportion of cell wall thickening in September was greater for the branches than for the stem, as previously observed in 2017 (**Figures 6C, F**). The progression of leaf senescence was similar in both years (**Figures 6A, D**).

## 4 Discussion

### 4.1 Meteorological conditions and wood growth cessation timing

Our dataset on the progression of xylem cell wall thickening in the second half of the growing season, encompassing five years of data for stems in two species and one or two year for branches for four species, is unique for angiosperm tree species and, as such, is of high value as a reference and for any meta-analysis or modeling work (Friend et al., 2019; Silvestro et al., 2025).

Although the short time series limited our ability to perform a multivariate analysis of the potential drivers of wood growth cessation, thereby hindering the investigation of confounding effects, our univariate analysis still reveals a strong correlation between the timing of xylem growth cessation and climatic conditions during the growing season. Our findings show that high August maximum temperatures strongly advanced the timing of wood growth cessation for both birch and beech. A potential mechanistic explanation is that elevated temperatures accelerate lignification and cellulose deposition in xylem cell walls, shortening the phase of secondary cell wall thickening (Crivellaro et al., 2022; Pérez-de-Lis et al., 2024). This pattern is clearly illustrated when comparing the cool year 2021 to the warm year 2020. In 2021, cell wall thickening was decline was slower, extending the growing season by approximately a month and a half. Considering that thickening typically starts in mid-May, this extension corresponds to a 30% increase in thickening duration and carbon sequestration, independent of species.

It is also possible that higher temperatures had an indirect impact on xylem growth cessation through their interaction with the water cycle. In fact, soil drought (SPEI) for beech and air dryness (VPD) and soil water content (SWC) for birch showed similar or even stronger correlations with the timing of xylem growth cessation than temperature. For the late-successional beech, sensitivity of wood formation to drought has been documented across Europe, with an overall long-term (multiannual) decrease in wood production in drier areas, but intra-annual growth dynamic studies remain scarce (Martinez del Castillo et al., 2022; Gribbe et al., 2024). Notably, the severe soil drought during the summer of 2018 not only reduced growth but also delayed the peak of cell wall thickening, as evidenced by our first sampling date (D’Andrea et al., 2020; Schuldt et al., 2020). This was followed by a marked decline in thickening activity in beech (D’Andrea et al., 2020). An earlier cessation of growth may enhance reserve storage, serving as a preferential pathway when carbon availability is limited (Rezaie et al., 2023). For the pioneer birch, little large-scale and long-term growth information is available due to its low economic value in Western Europe. Birch is known to show flexible senescence across years due to its sensitivity to water scarcity (Oksanen, 2021; Mariën et al., 2022). Comparing warm years with different precipitation regimes (2018-2020), Dox et al. (2022b) highlighted the sensitivity of birch in Norway to water availability, with prolonged cell wall thickening in wet years (low VPD), a pattern that is also evident here. The lower VPD facilitate carbon dioxide influx in the leaf, increasing carbon availability and lengthening of growth (Oksanen et al., 2019). High VPD combined with ample water availability can also prolong growth and delay senescence (Oksanen, 2021). Increased transpiration due to high VPD and high soil water content increases mineral uptake from the soil beneficial for plant nutrition and growth (Oksanen et al., 2019). This is in agreement with the delayed senescence observed here and, when considering saplings with the same characteristics (size, age, and provenance), growing under the same substrate conditions (soil volume, soil type, fertilizer amount, etc.), the ample irrigation during the two years, atmospheric dryness, and high temperature may collectively explain the higher rate of cell wall thickening in 2018. These results highlight the need for longer time series to evidence the dominant drivers and experimental work to disentangle the confounding factor of wood formation, particularly the combination of temperature, soil drought, and atmospheric drought (Buttò et al., 2025).

For both tree species, we found a spring legacy effect with warmer April (the period when cambial activity is reactivated advancing the xylem growth cessation in autumn (Marchand et al., 2021). An earlier start of wood growth is common in warmer springs (Prislan et al., 2019; Dox et al., 2022b). There is an ongoing debate about whether the start and end of the growing season covary for wood growth (Chen et al., 2022; Silvestro et al., 2025) but also for the leaf (Zohner et al., 2023; Zuccarini et al., 2025). One possible explanation is that sink limitation, referring to the demand for carbon by growth, directly regulates the plant’s carbon source by causally downregulating overall photosynthetic activity (Gessler and Zweifel, 2024). Following this theory, wood growth will cease early if wood growth starts early or if its rate is higher over the season. The sink limitation theory is in line with our results, with faster lignification of cell walls under warmer conditions, which may contribute to an earlier end of the growing season. Our results underscore the need for additional studies to uncover the mechanisms underlying variation in spring and autumn phenology, especially on longer time series of different angiosperm species.

### 4.2 Synchrony of stem and branch cell wall thickening in late season

Contrary to our hypothesis (ii), we found that branches and stems largely exhibited synchronized declines in cell wall thickening during late summer and autumn in both 2017 and 2018, across mature birch and beech trees as well as saplings of beech, birch, oak, and poplar. However, for birch and beech, for both saplings and mature trees, we observed occasionally but significantly higher cell wall thickening rates in branches and stems in early autumn. This prolongation of branch xylogenesis could be related to the fact that some branches maintain a minimal cambial activity until late in the season, to store the resources relocated during senescence (Barbaroux et al., 2003; Zahnd et al., 2024). The only exception to this was the more intense and delayed stem cell wall thickening of birch saplings in 2018. As mentioned above, this effect was maybe related to the high VPD and ample water content, increasing carbon and mineral acquisition, resulting in prolonged growth. In contrast to our findings, Gričar et al. (2017) showed that their model species, *Quercus pubescens*, presented earlier cessation of wood growth in branches than in the stem, but did not report the progression of cell wall thickening on a weekly scale, limiting the comparison. These authors speculated a potential influence of age on the different timing of autumn xylogenesis in branches and stem. A correlation between age and xylogenesis has also been emphasized in another ring-porous species, *Quercus robur* L (Marchand et al., 2021). Another mechanistic explanation is that ring-porous species, such as oak, are known to store more carbon reserves for the following year’s wood formation than diffuse-porous species like beech and birch. Toward the end of the growing season, they may prioritize reserve accumulation over growth, particularly in branches, which serve as major carbon storage compartments in the tree (Barbaroux et al., 2003).

## 5 Conclusion

Our results over five consecutive years show that spring and late summer temperature and water availability are important drivers of wood growth cessation for birch and beech. This study presents evidence of a general synchrony of xylem cell wall thickening in branches and stem during the late part of the growing season, but also the possibility of slightly prolonged branch growth in the absence of severe summer atmospheric drought. Our results will help modeling the aboveground forest growth of temperate angiosperm forests, and the relationship between aboveground growth and ecosystem functioning, under contrasting years and meteorological conditions. This is timely and especially relevant because wood phenology has been recently included in land surface models (Eckes-Shephard et al., 2022; Piponiot et al., 2022).

## Data Availability

The raw data supporting the conclusions of this article will be made available by the authors, without undue reservation.
