# Supplementary material for: Xylem growth cessation in stems and branches of European beech and silver birch: influences of temperature and drought
Source: Front Plant Sci. 2025 Aug 26;16:1648689. doi: 10.3389/fpls.2025.1648689 (PMC12418519; doi:10.3389/fpls.2025.1648689)
Supplement: Supplementary file 1 [file DataSheet1.docx]

Supplementary files:

**
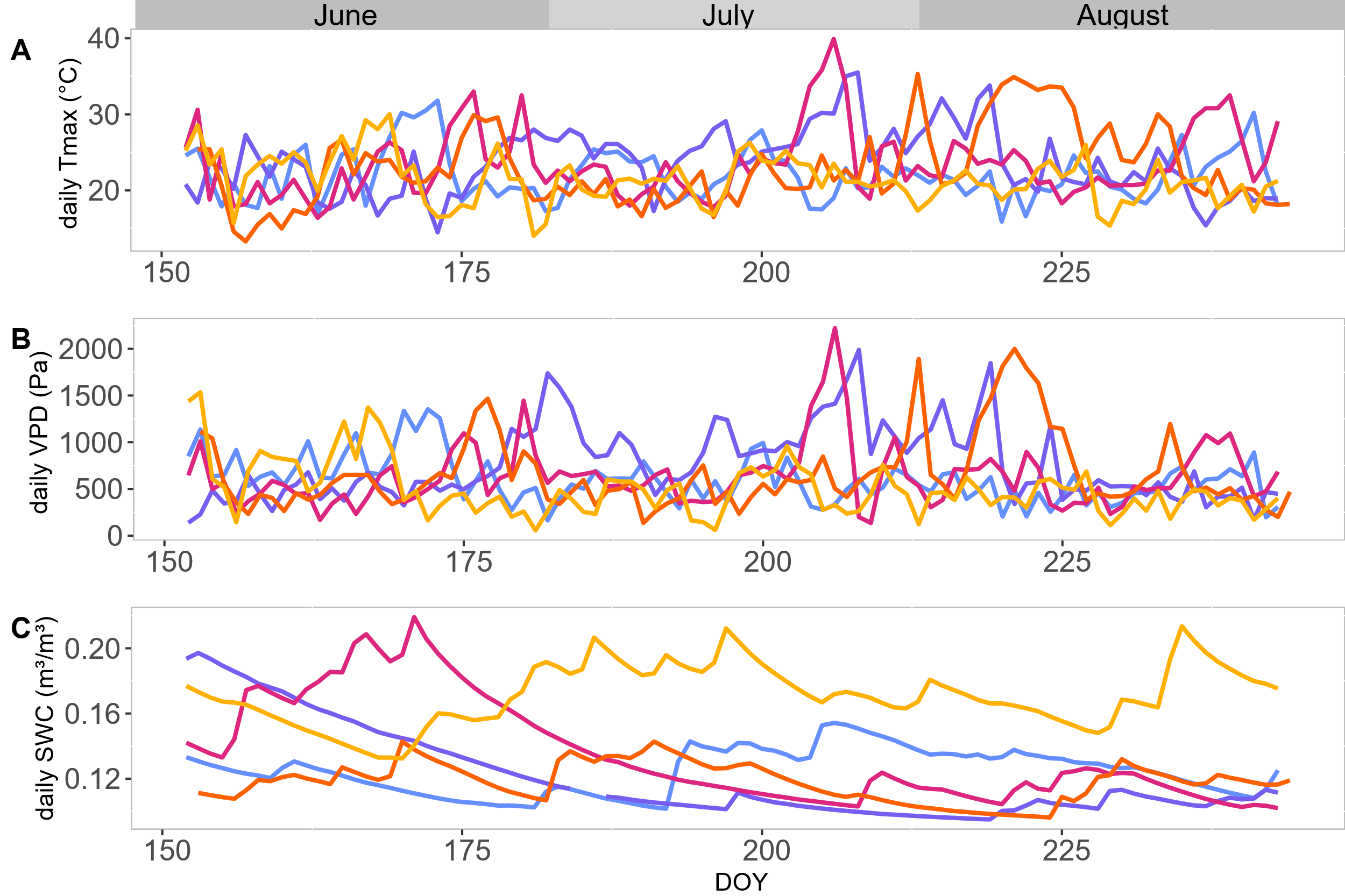
**

**Supplementary data Figure 1: Daily meteorological conditions between June and August for the period 2017-2021.** Soil water content (SWC) (m³/m³) at 0-80cm depth (A), maximum air temperature (Tmax) (°c) (B) and vapour pressure deficit (VPD) (Pa) (C) during June, July and August at Brasschaat over the five years studied. Color code: 2017 in blue, 2018 in purple, 2019 in pink, 2020 in orange and 2021 in yellow.


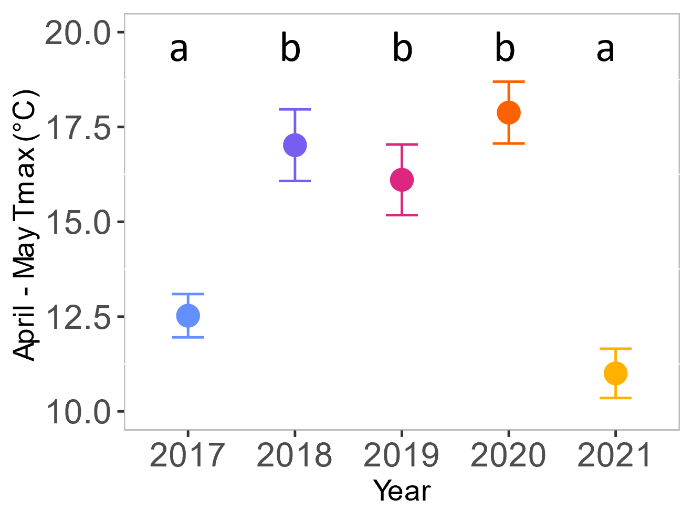


**Supplementary data Figure 2:** **Meteorological conditions for April and May for the period 2017-2021 at Brasschaat.** Daily maximal air temperature (Tmax; °C) during April and May at Brasschaat over the five years studied. Different letters indicate significant differences between years based on the Dunn test. Color code: 2017 in blue, 2018 in purple, 2019 in pink, 2020 in orange and 2021 in yellow.

**
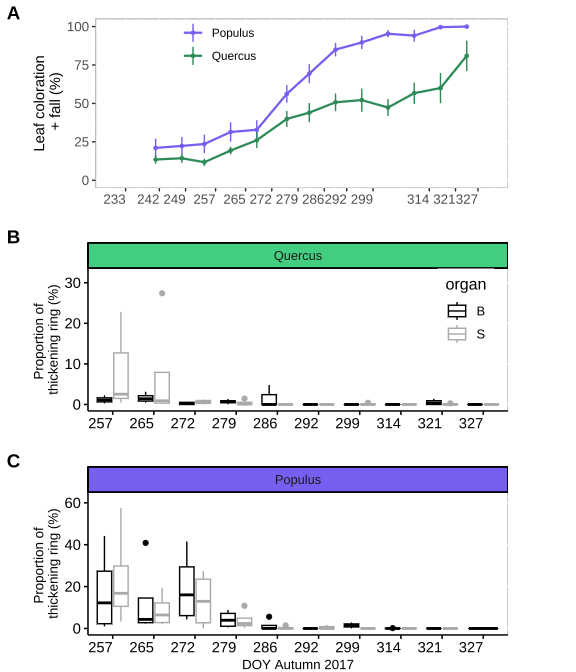
**

**Supp data Figure 3: Stem and branches xylem ring wall thickening in autumn 2017 for saplings of oak and aspen:** leaf senescence (proportion of yellow and fallen leaves) in autumn 2017 for saplings of pedunculate oak (*Quercus robur* L.) in green and common Aspen (*Populus tremula* L.) in purple (n = 12 sapling per species) (A). Proportion of xylem ring wall thickening for each sampling date in autumn 2017 for branches (black) and stem (grey) for saplings of oak (B) and aspen (C) (n=5 trees per species). The median (bold line) and interquartile range (box) are represented. vertical lines indicate the minimum and maximum values and dots indicate outliers. Significant differences between branches and stems based on Fligner Policello test are presented; ‘*’ for p-value<0.05, ‘**’ for p-value < 0.01 and ‘***’ for p-value < 0.001.

**Supplementary Table 1:** Day of year (DOY) of stem growth cessation (mean ± standard error) for mature Betula pendula and Fagus sylvatica trees.

| **DOY growth cessation** | **Betula** | **Fagus** |
| --- | --- | --- |
| 2017 | 270 ± 8 | 271 ± 0 |
| 2018 | 250 ± 5 | 272 ± 4 |
| 2019 | 261 ± 19 | 252 ± 3 |
| 2020 | 246 ± 8 | 256 ± 5 |
| 2021 | 293 ± 20 | 297 ± 7 |
